# A low-cost method to assess the epidemiological importance of individuals in controlling infectious disease outbreaks

**DOI:** 10.1186/1741-7015-11-35

**Published:** 2013-02-12

**Authors:** Timo Smieszek, Marcel Salathé

**Affiliations:** 1 Center for Infectious Disease Dynamics (CIDD), Department of Biology, The Pennsylvania State University, University Park, PA 16802, USA

**Keywords:** Sentinel surveillance, prevention, social network, influenza, collocation, SIR model

## Abstract

**Background:**

Infectious disease outbreaks in communities can be controlled by early detection and effective prevention measures. Assessing the relative importance of each individual community member with respect to these two processes requires detailed knowledge about the underlying social contact network on which the disease can spread. However, mapping social contact networks is typically too resource-intensive to be a practical possibility for most communities and institutions.

**Methods:**

Here, we describe a simple, low-cost method - called collocation ranking - to assess individual importance for early detection and targeted intervention strategies that are easily implementable in practice. The method is based on knowledge about individual collocation which is readily available in many community settings such as schools, offices, hospitals, and so on. We computationally validate our method in a school setting by comparing the outcome of the method against computational predictions based on outbreak simulations on an empirical high-resolution contact network. We compare collocation ranking to other methods for assessing the epidemiological importance of the members of a population. To this end, we select subpopulations of the school population by applying these assessment methods to the population and adding individuals to the subpopulation according to their individual rank. Then, we assess how suited these subpopulations are for early detection and targeted intervention strategies.

**Results:**

Likelihood and timing of infection during an outbreak are important features for early detection and targeted intervention strategies. Subpopulations selected by the collocation ranking method show a substantially higher average infection probability and an earlier onset of symptoms than randomly selected subpopulations. Furthermore, these subpopulations selected by the collocation ranking method were close to the optimum.

**Conclusions:**

Our results indicate that collocation ranking is a highly effective method to assess individual importance, providing critical low-cost information for the development of sentinel surveillance systems and prevention strategies.

## Background

Social network analysis has become an important tool to assess infectious disease spread in communities [1-3]. In a social network model of disease spread, the network ties between individuals are considered to be relevant for the transmission of the disease whose spread is modeled. For many infectious diseases, including some of the diseases with the greatest pandemic potential, such as influenza, disease transmission is assumed to require spatio-temporal proximity of individuals. Spatio-temporal proximity is typically approximated by network ties, where it is assumed that social contacts (family, friends, co-workers, and so on) capture the majority of potential disease transmission events.

The predictive power of social network topology on the dynamics of infectious disease spread has been confirmed empirically [1,3-6]. It is well established that both high individual contact rates as well as a high dispersion in population's contact distribution results in increased disease transmission within the population [7-9]. In many host-pathogen systems, it is a minority of individuals that contributes the most to infectious disease spread [8]. In addition, the position of individuals in a social network has been shown to correlate well with the likelihood and timing of infectious disease onset [1,3,10].

These findings immediately suggest an important role for social network analysis in the development of sentinel surveillance systems and targeted mitigation strategies. Sentinel surveillance is most efficient when disease outbreaks can be detected as early as possible. Mitigation strategies such as targeted vaccination are most efficient when each unit of resource (for example, vaccination dose) leads to the maximal case count reduction possible. Combined, these two methods can significantly mitigate infectious disease spread and thus reduce the morbidity and mortality associated with the disease.

Unfortunately, mapping social networks is very resource-intensive, and thus generally not a practical option in most communities. However, most communities do have information about the location of their members over time. For example, educational communities such as schools have detailed information about the location of their members in the form of rosters and schedules. From these readily available data sources, one can calculate the overall collocation time of each community member, that is, the cumulative time each individual is potentially exposed to other individuals. On a population-level, time-use surveys have been shown to be good proxies for contact data [11]. We suggest that also on a detailed community-level such a collocation measure can serve as a very good proxy indicator of the network measures that are associated with both increased infection likelihood and early infection during an outbreak. As a consequence, this method has the potential to be a simple, low-cost method to assess individual importance for early detection and targeted intervention strategies that are easily implementable in practice without the need to map social networks.

In this paper, we test how well collocation ranking can identify subpopulations suited for early detection and targeted intervention strategies. We compare the performance of the collocation ranking method (as defined by two benchmarks) to the performance achieved by other, partly network-based, methods. We further compare its performance to randomly selected subpopulations and the best possible subpopulations according to the two benchmarks.

## Methods

We challenge various indicators for selecting subpopulations for early detection and targeted intervention with computational influenza outbreak simulations that are based on empirical high-resolution contact and location data collected with wireless sensor technology at a US high school.

First, we describe the data that were used for our analyses. Then, we define two benchmarks according to which the proposed collocation ranking method and the other indicators are evaluated. Next, we describe all indicators that are tested in this paper. Finally, we describe the outbreak simulation model and how the performance tests were set up.

Both the empirical data and the simulation model are described in detail elsewhere [12]. Therefore, both are only described briefly here.

All simulations and analyses were coded in and executed by Python (Version 2.7.2, 32-bit, Enthought Python Distribution). Figures were created with R (Version 2.13.0) and the ggplot2 library.

### Ethics statement

The data collection was approved by the Stanford University Institutional Review Board on 24 July 2009. Written informed consent was obtained from all participants.

### Contact and location data

The data that we use in this paper were collected at a US high school during one school day with wireless sensor technology. A total of 789 individuals or 94% of the school population, including students, teachers, and staff, participated in the study. The participants wore small wireless sensors (so-called motes) that detect and log radio signals broadcast by other nearby motes. We refer to the motes that were worn by participants as mobile motes. Further, stationary motes were attached to fixed locations throughout the school campus to keep track of the participants' locations. As a consequence, the dataset contains two types of records. Close proximity interactions (CPIs) are records that indicate two participating individuals standing face-to-face with a distance of less than three meters at a certain point in time. Location records are records that indicate the presence of an individual nearby a stationary mote (location information is at the level of rooms). A detailed description on how information and noise were separated in the data is provided in the supplementary material (see Additional file 1). Data were collected at time intervals of 20 seconds.

### Schedule data

In many communities, full individual schedule data (that is, the schedule of each individual) is readily available to community health services. During an outbreak, this data could be used to calculate the collocation rank for all community members. For various reasons, it was not possible to obtain full individual schedule data from the school, but it was possible to obtain aggregated schedule data. We then reconstructed individual schedule data from a combination of the mote data and the aggregated schedule data. The aggregated schedule data file contains the following information about each class taught at the school during the mote deployment: (i) who taught the class, (ii) the room in which the class was taught, (iii) the period of the class, and (iv) the number of students who were signed up for the class. The aggregated schedule data combined with the high resolution location data allows us to reconstruct individual schedules with high fidelity. The algorithm for matching aggregated schedule and individual location data is further described in the supplementary material (see Additional file 1).

### Benchmarks

The core idea is to identify simple indicators that allow the identification of subpopulations of the entire school population that are maximally relevant in prevention and surveillance efforts. Both prevention and surveillance efforts should target individuals who are more likely to become infected than others. In addition, these efforts should be targeted at individuals who become infected early during an outbreak, allowing for early detection of outbreaks (surveillance) and early response (prevention).

We define two simple benchmarks to test the accuracy of any indicator to be evaluated:

1. The first benchmark *B*_1 _is the average probability $\bar{P_{i}}$ of the individuals *i *of a subpopulation *S *to become infected. We use an empirical probability *P_i _*that is defined as the ratio of the number of simulation runs *n *in which individual *i *in subpopulation *S *became infected and the total number of simulation runs *N *. Note that simulation runs in which *i *is the index case are ignored (see test setting section below). A subpopulation has been optimally identified if *B*_1 _is maximal.

2. For the second benchmark, we calculate the ratio $\bar{t_{i}}/P_{i}$ for every individual *i *in subpopulation *S *, where $\bar{t_{i}}$ is the average simulation time step at which the individual became symptomatic. Then, the second benchmark *B*_2 _is defined as the average of these ratios. The division by *P_i _*is necessary to take into account that early detection of a symptomatic individual is more relevant when the infection probability of that individual is high. The time of the onset of symptoms has more practical relevance than the time of infection, because symptomatic cases can be identified more easily than pre-or asymptomatic carriers. A subpopulation has been optimally identified if *B*_2 _is minimal.

### Rank indicators

Several indicators are evaluated with respect to their ability to select subpopulations with optimal benchmark results. Thus, a good indicator would select subpopulations that have high *B*_1 _values and low *B*_2 _values. The basic principle of subpopulation formation is the same for all indicators: the individuals are ranked according to their individual respective indicator value (from high to low values). Then, subpopulations are formed by selecting individuals from high to low ranks until the target subpopulation size is reached. We use the following rank indicators:

#### Presence

The presence indicator measures the total time an individual attends classes according to the schedule, and it is defined as $\overset{7}{\underset{p=1}{\sum }}t\left(p\right)\;\cdot T\left(p,i\right)$, where *p *is an index pointing to one of the seven periods of the surveyed high school day, *t*(*p*) is the official duration of period *p*, and *T*(*p,i*)=1 if individual *i *had a scheduled class during period *p *, and *T*(*p,i*)=0 if not.

#### Collocation

The collocation indicator measures the cumulative time each individual is potentially exposed to other individuals during classes, and it is defined as $\overset{7}{\underset{p=1}{\sum }}t\left(p\right)\cdot \omega \left(p,i\right)$. Here, *ω*(*p*,*i*) denotes the number of students signed up for the class that individual *i *is taking during period *p *, and *t*(*p*) is the official duration of period *p*. If *i *has no class during that period, *ω*(*p_,_i*)=0. The collocation indicator - like the presence indicator - is only based on schedule data.

#### Degree

We use the actor degree centrality *C_D_*(*i*) [13], which is one of the network indices that is frequently used in network epidemiology to identify the most important individuals in a transmission network [12,14-18]. The actor degree centrality of an individual *i *is defined as the number of contact partners of *i *- here determined by the presence of at least one CPI - during the measurement period.

#### Degree (>10 minutes)

The difference between this indicator and the previous one is that only contacts of more than 10 minutes of accumulated duration during the measurement period are considered. The cut-off of 10 minutes was chosen arbitrarily, but a sensitivity analysis shows that the indicator's performance changes only slightly when the cut-off is changed to 5, 15, or 20 minutes (see Additional file 1).

#### Strength

The strength of an individual *i *stands for the cumulative contact duration of *i*, and it is defined as $\underset{{}_{j}\in_{J}\Delta_{\left\{i\right\}}}{\sum }w\left(i,j\right)$. Thereby, *_J_*Δ_{*i*} _is the set containing the entire school population except *i *, and *w*(*i*,*j*) stands for the accumulated contact duration of individuals *i *and *j*. Strength is an enhancement of the degree concept and can be interpreted as a weighted degree [19].

There are other network measures which are frequently used to identify pivotal individuals in a social network, for instance, closeness centrality or betweenness centrality. These measures, however, have been shown to be comparably good or even worse than the degree in indicating individuals who are important for disease spread [17,18]. For this reason we concentrate on the simpler, but still powerful, centrality indicators described above.

### Model of influenza spread

We use an individual-based model of influenza spread to assess the importance of the members of the school population with respect to disease spread. The model is published and described in detail in [12], but briefly, we assume that the infection is introduced by one index case at the beginning of a simulation run and that all subsequent infections happen within the school population, that is, there are no further introductions from outside. The time step duration is half a day. The model is a susceptible, exposed, infectious, recovered (SEIR)-type model. The probability to switch from the susceptible to the exposed state is 1-(1-0.003)*^w^*, where *w *is the accumulated contact time the susceptible individual has spent with infectious individuals while at school (in CPI records) [20]. The duration of the exposed state follows a Weibull distribution with an offset of half a day; the power parameter is 2.21, the scale parameter is 1.10 [21]. After that period in the exposed state, every individual will be in the infectious state for exactly one time step before turning into home confinement and, finally, recovering. To allow for the fact that the onset of influenza symptoms is typically sudden and that affected individuals will be dismissed quickly, we reduce the duration of contacts by 75% during the single time step at school.

### Test setting

Each member of the school population could be the index case of an outbreak and introduce the infection from outside the school. Therefore, we initialize 100 independent runs for each member of the school population being the index case that introduces the infection. This results in a total of 78,900 simulation runs that build the basis of our analyses.

For all simulation runs, we keep track of which individuals got infected and when they became symptomatic during the course of the simulation run. This allows us to calculate the two benchmarks defined above.

## Results

In order to assess the performance of the collocation indicator, we selected subpopulations of various sizes on the basis of the collocation indicator and compared their benchmarks to randomly selected subpopulations, optimal subpopulations, and subpopulations selected on the basis of the other indicators described in the Methods section. An optimal subpopulation stands for a subpopulation selected in such a way that it achieves the best possible benchmark value for the given population.

### First benchmark: average infection probability

**Figure 1:**
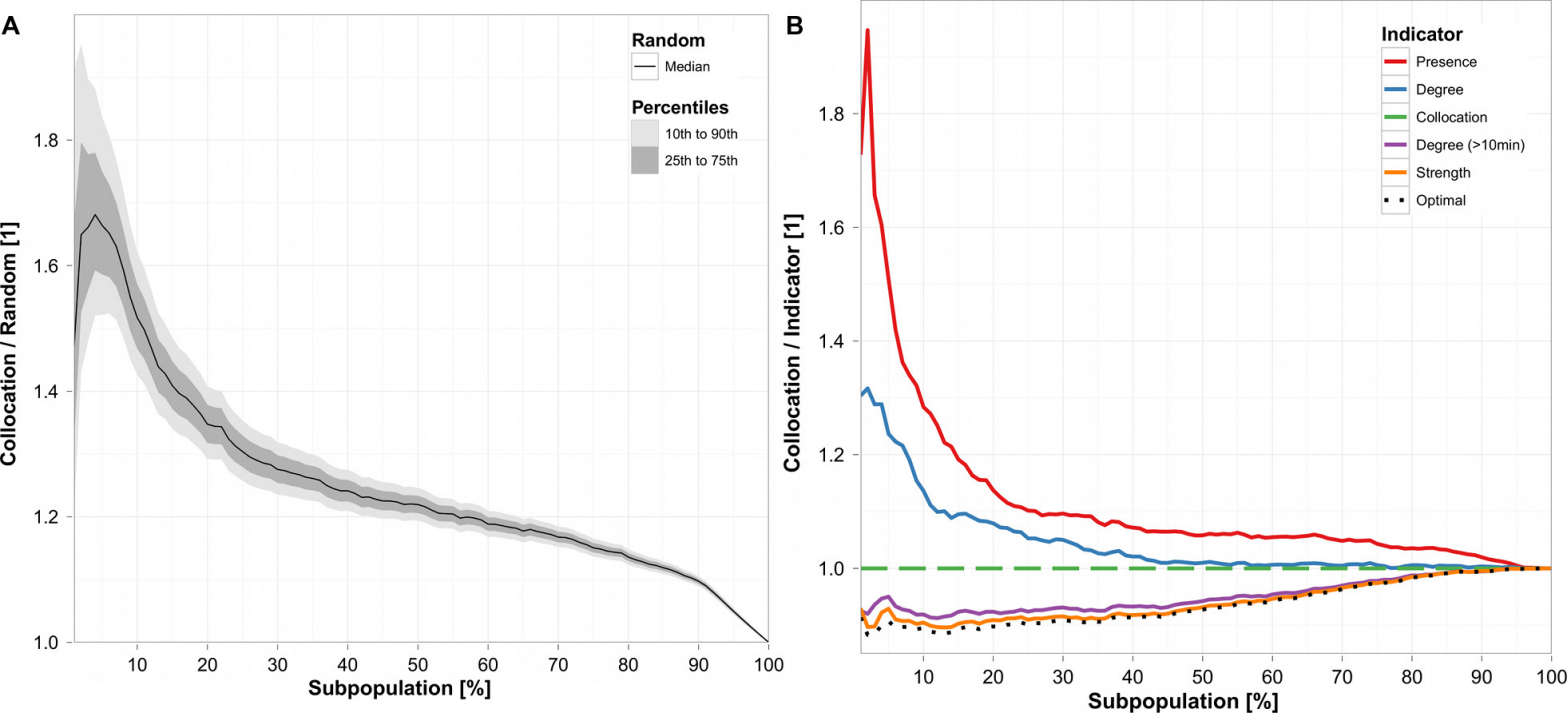
**Performance of collocation ranking: first benchmark**. Subfigures 1a and 1b are based on the first benchmark, which is the average probability of individuals in a given subpopulation to become infected during an outbreak, *B*_1_. The abscissa shows the percentage of the population selected for prevention or surveillance efforts. The ordinate shows the ratio of the *B*_1 _of the collocation indicator and the *B*_1 _of any other indicator, that is, ordinate values >1 indicate that the collocation indicator performs better than the other indicator it is compared to. Subfigure 1a compares the *B*_1 _value of the 10th, 25th, 50th, 75th, and 90th percentile of 100,000 randomly selected subpopulations to the *B*_1 _of subpopulations selected by the collocation indiciator. Subfigure 1b compares *B*_1 _of all indicators defined in the Methods section, as well as the optimal *B*_1 _, to the *B*_1 _of the collocation indicator.

The subpopulations that were selected on the basis of the collocation indicator constantly show a substantially higher average infection probability *B*_1 _than randomly selected subpopulations of the same size (Figure 1a). Given a subpopulation of ten percent of the entire school population, collocation ranking resulted in 1.43 to 1.62 times better results than randomly composed subpopulations that were between the 10th and the 90th percentile. Given a subpopulation of twenty percent of the entire school population, collocation ranking resulted in 1.29 to 1.41 times better results than randomly composed subpopulations that were between the 10th and the 90th percentile.

Most subpopulations selected on the basis of rank indicators achieved consistently better benchmark results than random subpopulations, and all of them outcompeted random composition over a large range of subpopulation sizes. The performance of subpopulations selected on the basis of the collocation indicator was better than the performance of subpopulations selected on the basis of the presence and the degree indicator, but worse than the performance of subpopulations selected on the basis of the degree (>10 minutes) and the strength indicator (Figure 1b). For subpopulations smaller than 40% of the entire population, those selected on the basis of the collocation indicator achieved benchmark values that were only about 10% below the optimum.

### Second benchmark: ratio of average infection time and probability

The qualitative picture for the second benchmark was very similar to that of the first benchmark. However, differences between the various subpopulations were more pronounced.

**Figure 2:**
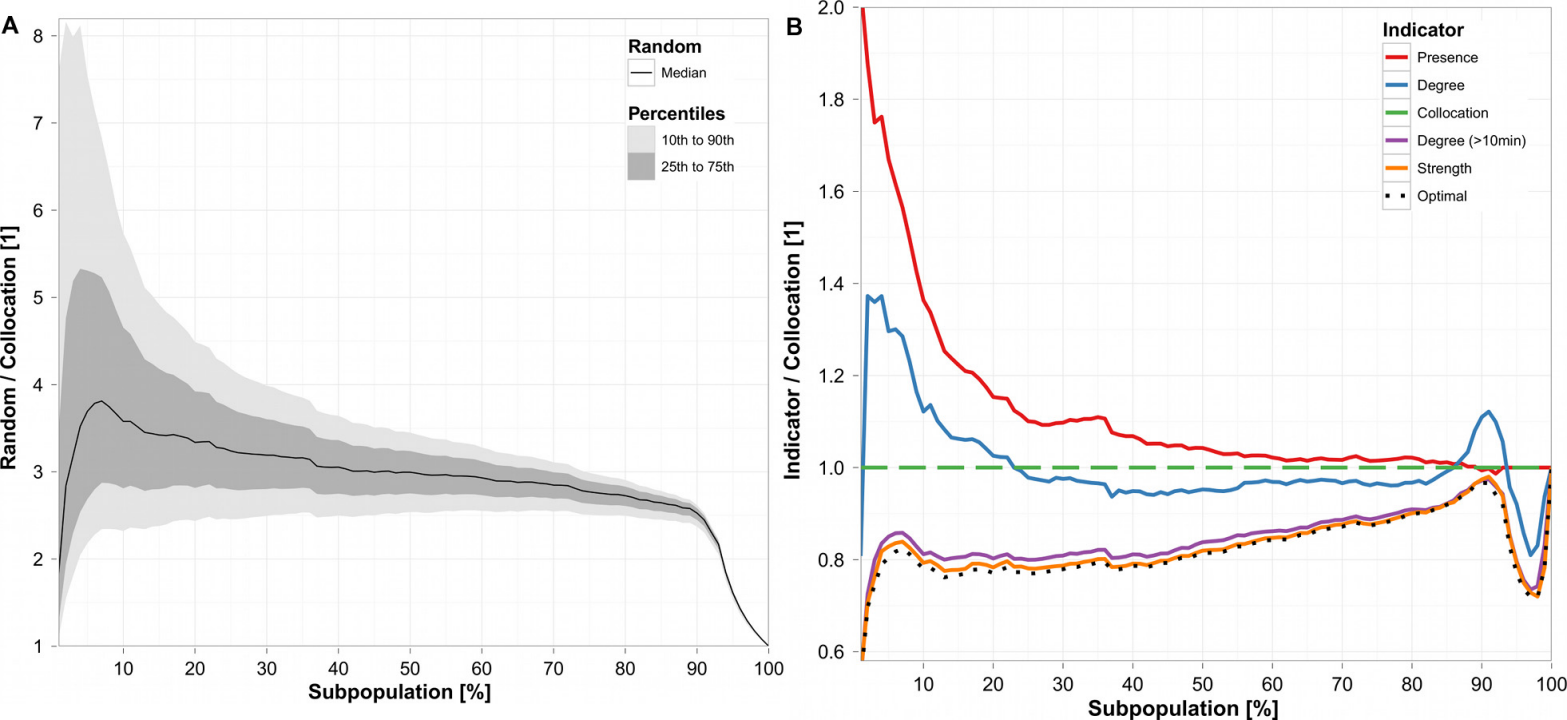
**Performance of collocation ranking: second benchmark**. Subfigures 2a and 2b are based on the second benchmark. The abscissa shows the percentage of the population selected for prevention or surveillance efforts. The ordinate shows the ratio of the *B*_2 _of a given indicator and the *B*_2 _of the collocation indicator, that is, ordinate values >1 indicate that the collocation indicator performs better than the other indicator it is compared to. Subfigure 2a compares the *B*_2 _value of the 10th, 25th, 50th, 75th, and 90th percentile of 100,000 randomly selected subpopulations to the *B*_2 _of subpopulations selected by the collocation indicator. Subfigure 2b compares *B*_2 _of all indicators defined in the Methods section, as well as the optimal *B*_2_, to the *B*_2 _of the collocation indicator.

For subpopulations that represent between 2% and 90% of the entire population, the subpopulation selected on the basis of the collocation indicator performed consistently 2.5 or more times better than the median of the random subpopulations. For almost the entire range of subpopulation sizes, the benchmarks of the subpopulation selected on the basis of the collocation indicator were at least twice as low as the benchmarks of 90% of the random subpopulations (Figure 2a).

For small subpopulations up to approximately 22%, collocation ranking outcompeted ranking by degree. Further, collocation ranking was almost always better or as good as ranking by presence (Figure 2b).

### Further analyses

Additional analyses, in particular how the role of members of the school population (that is, whether the individual is a student, a teacher, or a staff member) is related to individual importance, are provided in Additional file 1.

## Discussion

Social networks have proven to be useful for understanding and predicting infectious disease dynamics. There is a discussion on how detailed network data must be in order to be useful in epidemiological applications [6,14,22]. However, even mapping low-detail social contact networks is typically too resource-intensive to be a practical possibility for most communities and institutions. What is needed instead are low-cost proxies for individual network properties that can serve as epidemiological predictors. Spatial distance measures, for example, have recently been found to be significant predictors of social ties (among other predictors) [23], and it is therefore reasonable to expect that spatial proxies can also serve as useful epidemiological predictors. The collocation ranking method presented here is based on spatio-temporal considerations, and our results suggest that it may effectively identify subpopulations suited for sentinel surveillance systems and prevention strategies.

Current methods to identify subpopulations for sentinel surveillance systems and prevention strategies typically rely on demographic variables such as age (for example, children and young adults in influenza surveillance systems [24-26]) and geographic location (for example, administrative units in invasive meningococcal disease surveillance systems [27-29]). These methods work because there is sufficient variance of such demographic variables at the societal level. However, at the level of communities and institutions such as schools, there is often too little variance to make these methods applicable. Furthermore, because demographic variables are not direct proxies for transmission routes, they may fail to identify individuals with high transmission potential who fall outside of the targeted range of the demographic variable. In contrast, the collocation ranking indicator proposed here is a direct proxy of potential disease transmission events as given by the contact network.

Random selection serves as a null model method in the absence of epidemiologically relevant information about a population. The collocation ranking method significantly outcompetes the random method. As expected, some network indicators, such as the strength, were able to outcompete the collocation ranking method to identify subpopulations for early detection or targeted intervention strategies. This is not surprising because strength is essentially a direct measure of exposure, and it can thus serve as an indicator that can identify subpopulations which are almost identical to the optimal subpopulation. Nevertheless, measuring strength is resource-intensive, while collocation ranking is not.

Our research is not without limitations. The first limitation is that we rely on widely used computational simulation models of disease spread, rather than validating our method in an empirical setting. Our simulation model is based on high-resolution contact network data [12] as well as established disease transmission parameters [20,21], but ideally, any benchmark would be based on empirical outbreak data instead of simulated data. However, infection transmission is a highly stochastic process, requiring multiple outbreaks for a robust evaluation of the collocation ranking method presented above.

Limitations and uncertainties of our model are, in particular, the following: (i) There is still debate on the relative importance of the different potential pathways of influenza transmission [30-32]. Most models of influenza spread assume transmission by close contact, but there is the possibility that other transmission pathways are more important than currently thought. (ii) We model the spread between members of the school population during school hours, but we do not capture potentially infectious contacts between school members during their leisure time. (iii) We assumed that the probability of being an index case is homogeneous. In reality, this is most likely not the case. (iv) We also assumed that all individuals are fully susceptible. In reality, individuals differ in their serostatus and (partial) immunity is linked to patterns of previous exposure. (v) It might be that an ongoing epidemic changes the contact behavior not only of the symptomatic individuals, but also of the healthy ones who continue to attend school. Such potential behavior changes are not reflected in our model.

Another limitation is that the data to test our method were collected in one school only. Moreover, the data covers only one school day. While the method worked very well in this setting, the generalizability to other settings remains to be established.

Finally, we had to reconstruct individual schedules from aggregated schedules and mote data. Reconstructions may be incomplete (compare with Additional file 1), and the real course of a school day may differ from the scheduled sequence of classes. While it is important to recognize that we currently cannot conclusively validate our method, our simulation results indicate that the collocation method is an effective, low-cost tool that warrants further research.

## Conclusions

Social networks have proven to be useful predictors of infectious disease outbreak dynamics. From a practical perspective, social network information can be highly valuable for the development of sentinel surveillance systems and prevention strategies because people's positions within the network correlate with their likelihood and timing of infection during an outbreak. The disadvantage of network-based approaches is that they are highly resource-intensive and, thus, can not be applied to every situation of interest. Hence, simple proxies, such as the collocation ranking method presented here, that fulfill the same purpose are needed. Subpopulations identified by the collocation ranking method are significantly better suited for sentinel surveillance systems and prevention strategies than randomly selected subpopulations. Some network-based ranking methods were slightly better for identifying such subpopulations than collocation ranking. The collocation ranking method, however, is a low-cost method that still manages to identify subpopulations that are very close to the optimum. The data requirement of the method is very low, and typically readily available in many community settings, such as schools, offices, hospitals, and so on in the form of rosters/schedules.

Our results suggest that the collocation ranking method may effectively identify subpopulations suited for sentinel surveillance systems and prevention strategies.

## Abbreviations

CPI: close proximity interaction; SEIR: susceptible, exposed, infectious, recovered.

## Competing interests

The authors declare that they have no competing interests.

## Authors' contributions

TS and MS conceived and designed the study. MS collected the data. TS performed the analyses. TS and MS contributed to the writing of the manuscript. Both authors read and approved the final manuscript.

## Pre-publication history

The pre-publication history for this paper can be accessed here:

http://www.biomedcentral.com/1741-7015/11/35/prepub

## Supplementary Material

Additional file 1 **Supplementary information**. This additional file contains further information on (i) the data collection, (ii) how the locations of study participants were derived from the data, and (iii) how the individual schedules of students and teachers were reconstructed. The file further provides supplementary analyses which are not included in the main document. In particular, it contains figures that show (i) how well the five indicators define subpopulations according to a third benchmark (the average time to the onset of symptoms), (ii) how sensitive the outcome of the degree indicator reacts to various contact duration cut-offs, (iii) how predictive the role of an individual is for the likelihood and timing of infection, (iv) what the relationship between the five indicators is, and (v) how well the collocation indicator captures the number of infections that are induced by a certain index case. Click here for file
