# Supplementary material for: A low-cost method to assess the epidemiological importance of individuals in controlling infectious disease outbreaks
Source: BMC Med. 2013 Feb 12;11:35. doi: 10.1186/1741-7015-11-35 (PMC3616813; doi:10.1186/1741-7015-11-35)
Supplement: Additional file 1 — Supplementary information. This additional file contains further information on (i) the data collection, (ii) how the locations of study participants were derived from the data, and (iii) how the individual schedules of students and teachers were reconstructed. The file further provides supplementary analyses which are not included in the main document. In particular, it contains figures that show (i) how well the five indicators define subpopulations according to a third benchmark (the average time to the onset of symptoms), (ii) how sensitive the outcome of the degree indicator reacts to various contact duration cut-offs, (iii) how predictive the role of an individual is for the likelihood and timing of infection, (iv) what the relationship between the five indicators is, and (v) how well the collocation indicator captures the number of infections that are induced by a certain index case. [file 1741-7015-11-35-S1.PDF]

Supplementary Information

Smieszek T, Salathé M. A low-cost method to assess the epidemiological importance of individuals in controlling infectious disease outbreaks.

Code and data availability

Both the code and the data are publicly available.

Collection of contact and location data

A detailed description of the data collection is already published elsewhere [1]. Here, we only recapitulate the most important facts and add information that goes beyond the scope of the previous publication.

On January 14, 2010, wireless sensor network motes were distributed to the population of an American high school, including students, teachers, and staff. Motes were placed in a thin plastic pouch attached to a lanyard and worn around the participants' neck throughout the entire school day. We refer to motes worn by the participants as ***mobile motes***. 94% of the school population agreed to participate and activated the motes correctly so that they logged data. Motes were also placed at fixed locations throughout the school building to be able to track the location of the mobile motes over time. We refer to those fixed motes as ***stationary motes***.

Both mobile and stationary motes broadcast packages of data (including the motes' IDs) at an interval of 20 seconds. The power of the signals was programmed to be much stronger for the stationary motes (-11.0 dBm) than for the mobile motes (-16.9 dBm). The data packages were received and logged solely by mobile motes (Figure S1). Received mobile mote signals were filtered according to signal strength to make sure that only face-to-face contacts of up to three meters distance were logged. We refer to such a contact as ***close proximity interaction (CPI)***.

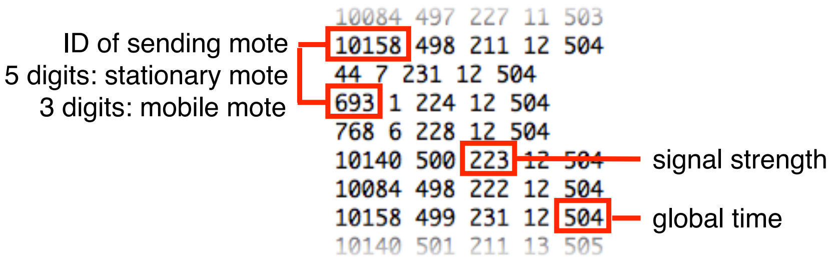

Fig. S1: Extract from raw data downloaded from a mobile mote. Mobile motes received and logged signals from other mobile (3 digit ID, first column) or stationary (5 digit ID, first column) motes. Each row represents one signal from one particular mote at a particular time step. The signal strength of the received signal is given as a manufacturer-specific code that can be transformed into RSSI or dBm values (third column). One unit in the global time (fifth) column equals 20 seconds.

At a given global time t, a mobile mote can receive multiple signals (CPIs) stemming from different other mobile motes (Figure S1). A CPI is, by definition, always mutual: If i had face-to-face contact with j, then j also had face-to-face contact with i. Sometimes, only one of the two motes that form a CPI detected the other mote's signal. Thus, we had to make sure that for every arc from individuals i to individual j at time t, $< i, j >$, there is also an arc $< j, i >$.

At a given global time t, a mobile mote can also receive multiple signals from stationary motes. In an ideal situation, the strongest of all signals received from stationary motes at time t would indicate the location of the individual that wore the mobile mote. However, real-world settings, such as the school where the data was collected, are rarely ideal, and, hence, the collected location data is expected to be noisy.

Identification of participant locations

There were several sources of noise and bias that affected signals received from stationary motes: (i) wireless local area network (WLAN) stations and other sources of electromagnetic waves that interfered with the signals from the motes; (ii) disadvantageous placements of motes that were oftentimes unavoidable because the stationary motes had to be placed at secure places, e.g., on cabinets or bookshelves; (iii) uncontrollable refraction, reflection, and signal dampening caused by walls and objects.

In order to reduce the noise in the location data, we modified the data by employing a very conservative approach (based on physical laws and additional knowledge on the operation of a school) where we assumed:

1) Individuals who are linked by a CPI at a given global time t were typically at the same location. In particular, if the location of one individual was a clearly enclosed space of limited size (e.g., a classroom as opposed to a long hallway or a cafeteria), it is physically impossible that the other, linked individual was in a different room.
2) The locations of individuals in a school are relatively stable during short periods of time (i.e., few time steps). Hence, when individuals appeared to oscillate between rooms with a high frequency according to our data, we assumed this to be an error.
3) Most of the teaching at a school takes place in classes, where a clearly defined group of individuals occupies one particular room in a building for a particular time during the day.

Based on these three assumptions, we defined and applied a sequence of four algorithms that reduced the noise in our location data. A comparison of the so modified data with the school's schedule on the deployment day indicated that the modified location data is a reasonably good representation of the school day (see section on the reconstruction of individual schedules).

Algorithm 1: Weighted average signals

The aim of the first algorithm was to determine initial locations for all individuals of the population and all time steps that are more robust than relying on the strongest signal. To do this, we first identified the set of all other individuals I that were linked to a specific individual i by a CPI at a specific time step t_0. Next, we calculated for each stationary mote that was detected at t_0 by at least one individual $j \in I \cup \{i\}$ the average detected signal strengths (in dBm) over all individuals in $I \cup \{i\}$. We repeated this procedure for the two time steps preceding t_0, t_{-2} and t_{-1}, as well as for the two subsequent time steps, t_1 and t_2. The resulting five sets of mean signal strength values were used to compute weighted signal strength averages over the five time steps t_{-2}, t_{-1}, t_0, t_1, and t_2. For the weighting, we used the following relative weights: $w(t_{-2}) = w(t_2) =$

1; $w(t_{-1}) = w(t_1) = 2$; $w(t_0) = 4$. The stationary mote with the highest weighted average signal strength determined the initial location of individual i at time t_0.

The information at time t_0 is usually the most accurate predictor for the actual location at time t_0. Therefore, the weight for t_0 was the highest. However, if the data at preceding and subsequent time steps congruently deviated from the data at t_0, then that information was able to overrule the information at t_0.

Algorithm 2: Smoothing

The aim of the second algorithm was to smooth interrupted sequences of locations. To this end, we identified all AABA (A stands for one location and B for another) and all ABAA subsequences in the data and replace them with AAAA subsequences. We based the smoothing algorithm on AABA and ABAA instead of ABA to make sure that A is the locally dominant location. Algorithms one and two improved the quality of the location data noticeably (Figure S2).

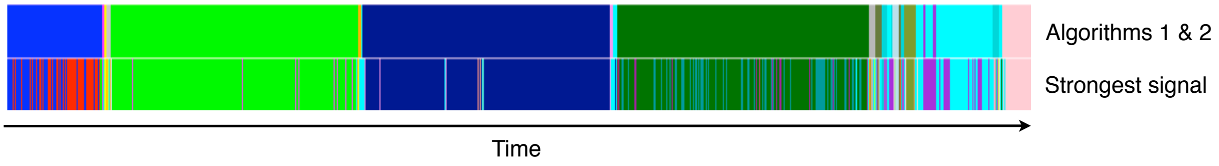

Fig. S2: An individual's locations over time. The color of the areas in both the upper and the lower bar indicate a particular location. The lower bar is based on the raw location data, where the strongest location signal record determines the color of an area increment. The upper bar shows the location allocation after applying algorithms one and two.

Algorithm 3: Consistent group locations

The aim of the third algorithm was to detect clearly defined and temporally stable groups of individuals and to make their location records consistent. Location records are inconsistent when different individuals belonging to one group have different locations.

First, we identified all pairs of stationary motes that met the following requirements: (i) they were involved in inconsistencies, (ii) at least one mote was placed in a classroom, and (iii) none of the motes was placed in the lunch break areas of the school. The reason for the explicit inclusion of classrooms was that the attendants of a class form a clearly defined group that exists for a prolonged time with typically a clear beginning and a clear end. The reasons for the exclusion of the lunch break areas were the open architecture of the schools' lunch break area and that students, teachers, and staff members move freely in that space.

Second, we identified for each such pair of stationary motes periods of time (at least 30 minutes) when temporally stable groups of individuals occupied the corresponding locations. We assumed that if (i) the total occupancy of both locations dropped below a certain threshold or (ii) the composition of the occupants changed substantially, the class period had ended.

We operationalized conditions (i) and (ii) as follows:

We defined two sets of individuals I_1 and I_2 as

$I_1 = I_{t-2} \cup I_{t-1} \cup I_t$ and

$I_2 = I_{t+1} \cup I_{t+2} \cup I_{t+3}$,

where I_t is the set of individuals allocated to one of the two stationary motes at time step t.

During a period, we expected the total occupancy of two adjacent rooms to be at least ten. If

$$|I_1 \cup I_2| \geq 10.$$

was not true, we defined condition (i) to be fulfilled.

We used the Jaccard index [2] to quantify the changes in the occupant composition. If

$$\frac{|I_1 \cap I_2|}{|I_1 \cup I_2|} \geq 0.5$$

was not true, we defined condition (ii) to be fulfilled.

Continuous periods of time of at least 30 minutes duration when neither condition (i) nor condition (ii) was fulfilled were stored. Both the thresholds for condition (i) and (ii) are arbitrary, but reasonable. The time periods that were generated by this algorithm corresponded almost perfectly to the bell times of the school.

Third, we defined networks of individuals for every pair of stationary motes and every time period (as defined in step two) and identified their connected components. An individual was a node of such a network, if it was allocated to one of the two stationary motes in more than half of the time steps of the period. Nodes of the network were linked, if they recorded CPIs between each other in more than half of the time steps of the time period.

We used the connected_components algorithm of the NetworkX 1.5 package for Python 2.7 (https://networkx.lanl.gov) to break down the so defined network into components. We ignored components of less than five nodes.

The fourth and final step depended on the number of components, n:

In the case of $n = 1$, we computed whether the nodes of this component were more often allocated to one stationary mote or to the other. Next, all entries of the inferior stationary mote were overwritten with the dominant one.

In the case of $n = 2$, one component was allocated to one mote and the other component to the other mote. The component that was more dominantly allocated to one of the motes triggered the concrete allocation of the two components.

In the case of $n > 2$, each component was allocated to the mote that was dominant for the respective component.

Algorithm 4: Consistent local neighborhoods

The aim of the fourth algorithm was to increase the consistency between location records of individuals and their neighbors when they did not belong to a group (as defined in the description of algorithm three). We identified for every individual i and every time step t to whom the individual was linked by a CPI at that time step t. We then checked whether a majority of these immediate neighbors was allocated to one specific stationary mote at t. If so and if the location of i differed, we adjusted the location of i. Algorithms three and four further increased the plausibility of the location data (Figure S3)

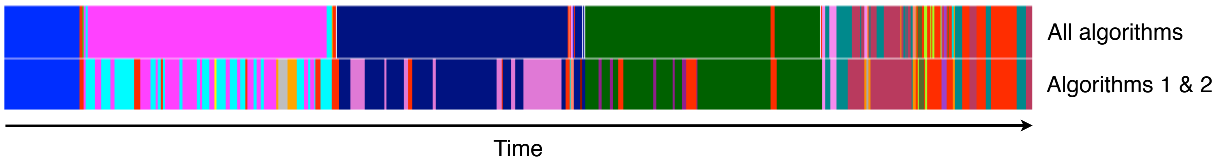

Fig. S3: An individual's locations over time. The color of the areas in both the upper and the lower bar indicate a particular location. The lower bar shows the location allocation after algorithms one and two were applied (see Figure S2). The upper bar shows the modified location allocation after all four algorithms were applied.

Properties of the modified location data

The extent of inconsistencies in the location data was substantially reduced after the four algorithms (described above) were applied (Figure S4). The total number of inconsistencies dropped to approximately 45% of the original value after applying algorithms one and two. All four algorithms decreased the number of inconsistencies to approximately 25% of the original value. During the periods the proportion of inconsistencies was typically about 5% or below. While the number of inconsistencies increased during breaks and lunch time, this is not necessarily a sign of a misrepresentation of reality since the school population spent these periods of time mostly in open areas where it is technically possible that one individual stood closer to one stationary mote and the other, linked individual stood closer to another mote.

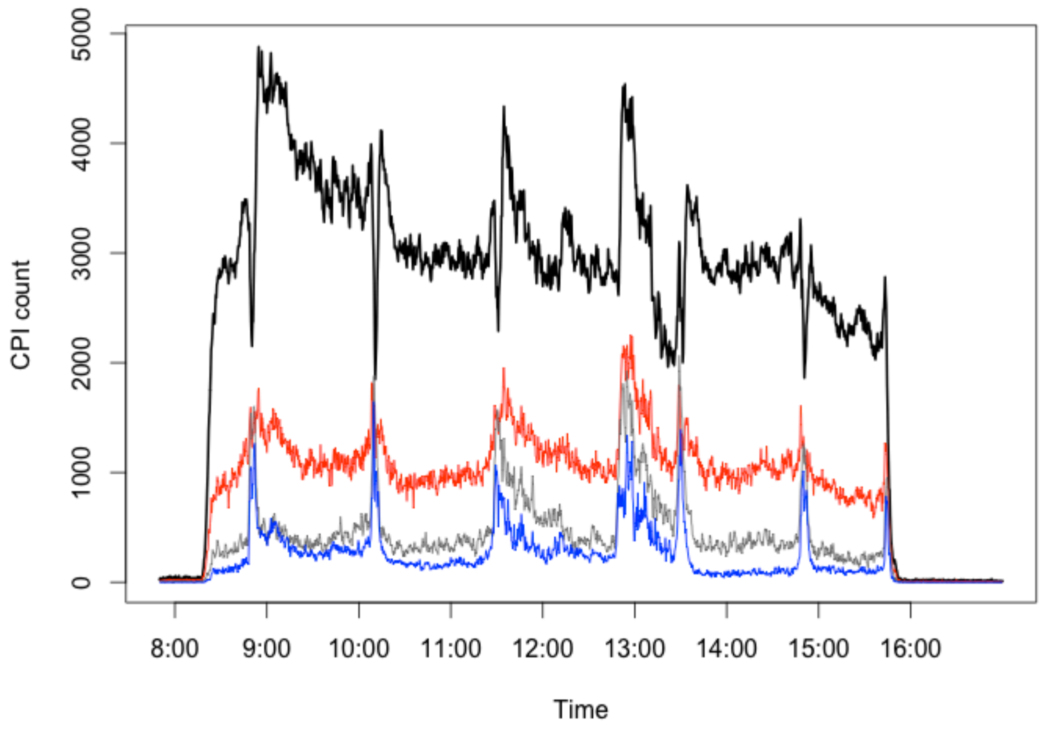

Fig. S4: Total number of CPIs over time (black line) as well as number of CPIs for which the two involved individuals are inconsistent with respect to location. The red line shows the number of inconsistencies if the strongest signal is used to determine the location; the grey line shows the number of inconsistencies for the location information after algorithms one and two were applied; the blue line shows the number of inconsistencies after all four algorithms were applied.

The modified location data corresponded well with the aggregated schedule of the school at the deployment day. This is further discussed in the subsequent section on the reconstruction of the schedules.

Reconstruction of individual schedules

The school provided aggregated schedule information, which contained the following information about each class taught at the school: (i) who taught the class, (ii) in which room the class was taught, (iii) the day and period of the class, and (iv) the number of students signed in.

With this information, the reconstruction of the individual schedules of the teachers was straightforward. The individual schedules of the students were reconstructed by combining aggregated schedule data and mote-based location data with the following algorithm:

1) The total number of students signed in to a class defined the (maximal) number of individuals that were to be assigned to the corresponding classroom. We refer to these places to be filled with individuals as empty slots.

2) Individuals were sorted from the highest to the lowest measured presence time in that classroom during the class' period. The empty slots were consecutively filled with individuals from this sorted list. An individual was assigned to an empty slot, (i) if it was next on the sorted list, and (ii) if there was no other room in which the individual spent more time during the period. An individual was not assigned to the classroom but flagged for further consideration if condition (ii) was not fulfilled.

3) Finally, we looped through all classes that had remaining empty slots and through all flagged individuals. If a flagged individual was not yet allocated to another, more dominant classroom, it was used to fill a remaining empty slot it fitted to.

With this algorithm, we were able to fill 84% of all empty slots. 70% of all classes could be filled completely. The difference between the aggregated schedule information and the reconstructed individual schedules can probably be explained to some extent by the remaining noise in the empirical location data. However, it has also to be taken into consideration that 6% of the school population did not participate in the study and, thus, did not contribute to the reconstructed occupancy data. Furthermore, 6.7% of all scheduled classes remained completely empty. This indicates that - at least some of them - were probably canceled.

Additional analyses not presented in the main text

Indicators versus average time of symptom onset

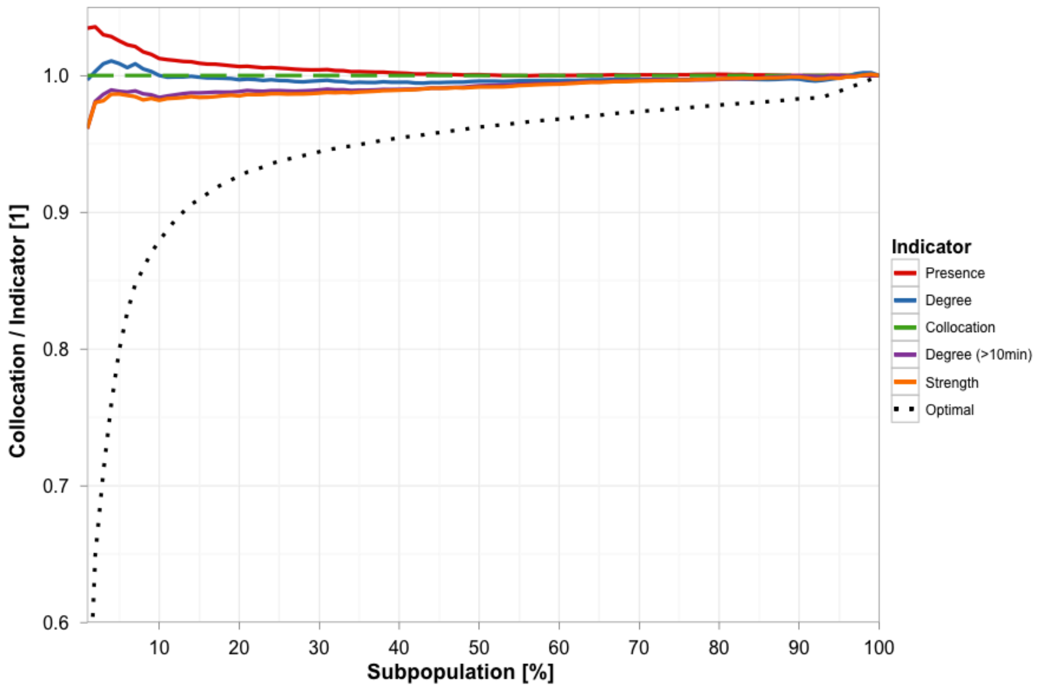

Fig. S5: This figure is analogous to subfigures 1b and 2b. Here, the average time to the onset of symptoms, $\bar{t}$, was chosen as the benchmark according to which all indicators are compared to the collocation indicator. Also shown is the optimal $\bar{t}$ relative to the collocation indicator.

Sensitivity analysis for the contact duration cut-off of the degree indicator

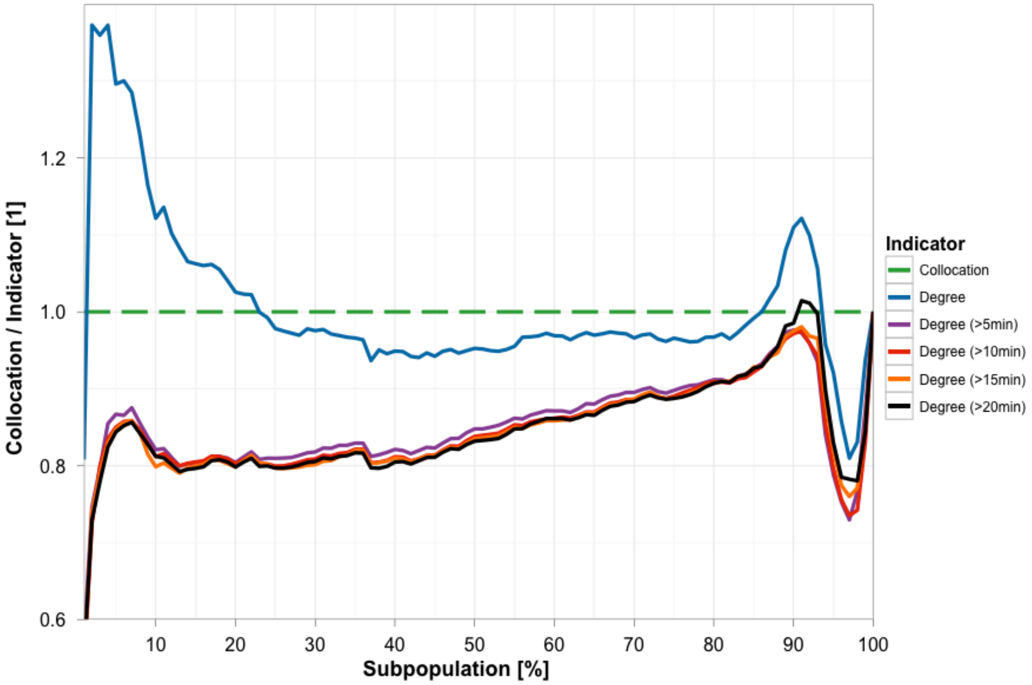

Fig. S6: This figure is analogous to subfigures 1b and 2b. It compares the performance (2nd benchmark) of the collocation indicator relative to several variations of the degree indicator: (i) the degree of the entire network, and the degrees of subnetworks that include only contacts with a duration of more than (ii) 5 minutes, (iii) 10 minutes, (iv) 15 minutes, (v) 20 minutes.

Role of individuals versus their epidemiological importance

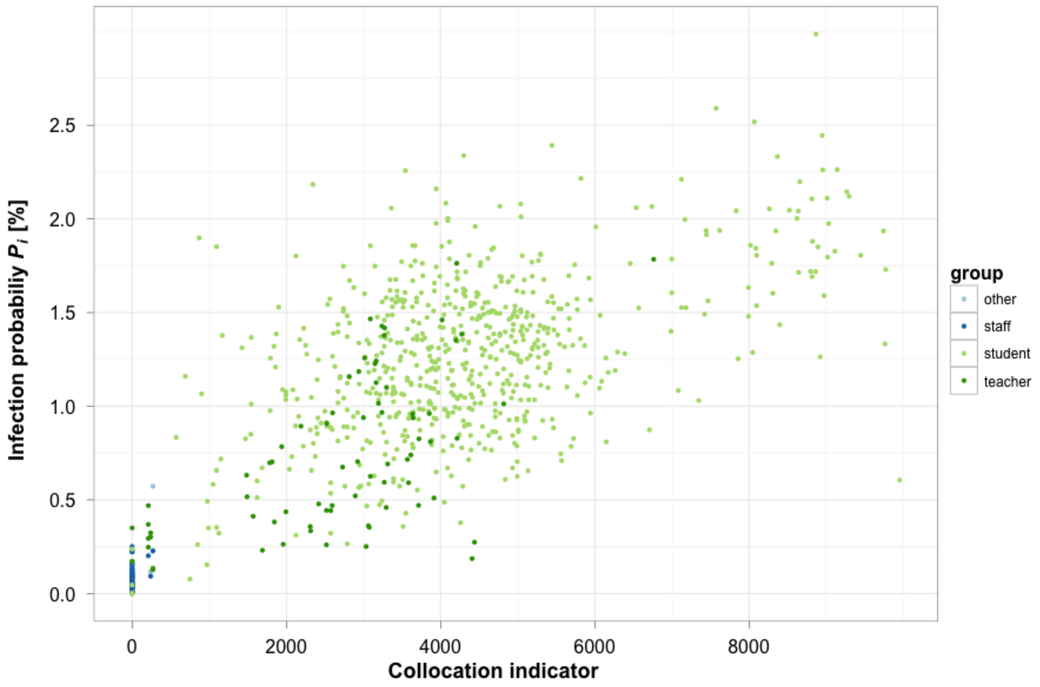

Fig. S7: Scatterplot of the individuals' collocation indicator values versus their probability of becoming infected during an outbreak. The color code signifies the role of each individual at the school.

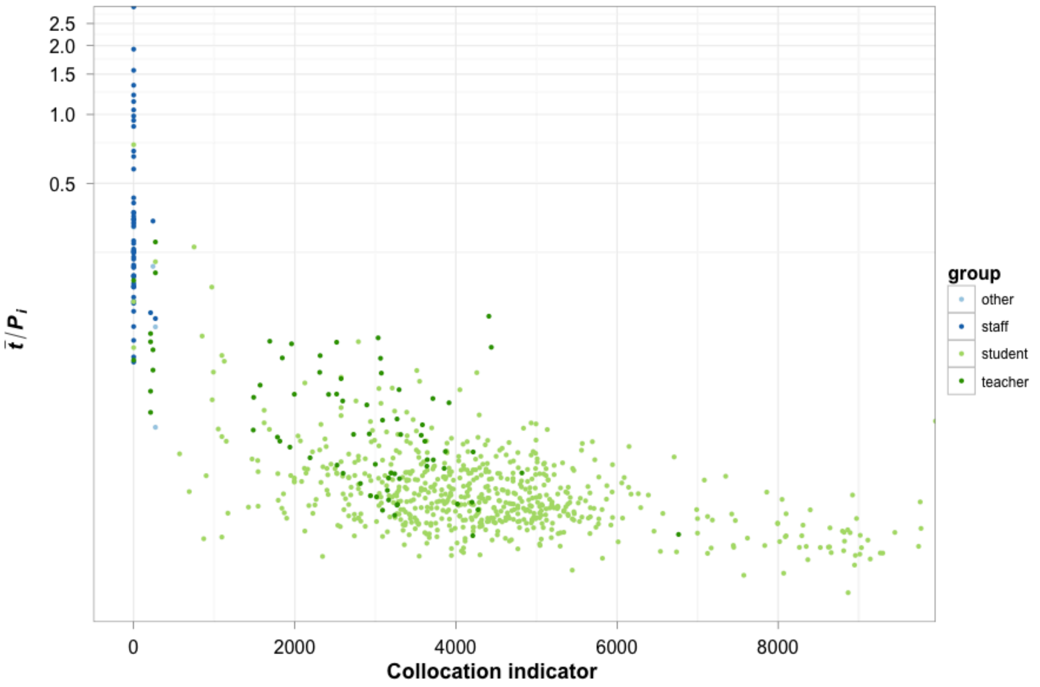

Fig. S8: Scatterplot of the individuals' collocation indicator values versus the ratio $\bar{t}/P_i$ *. The ordinate axis is logarithmic. The color code signifies the role of each individual at the school.*

Distributions of and relationships between the indicators

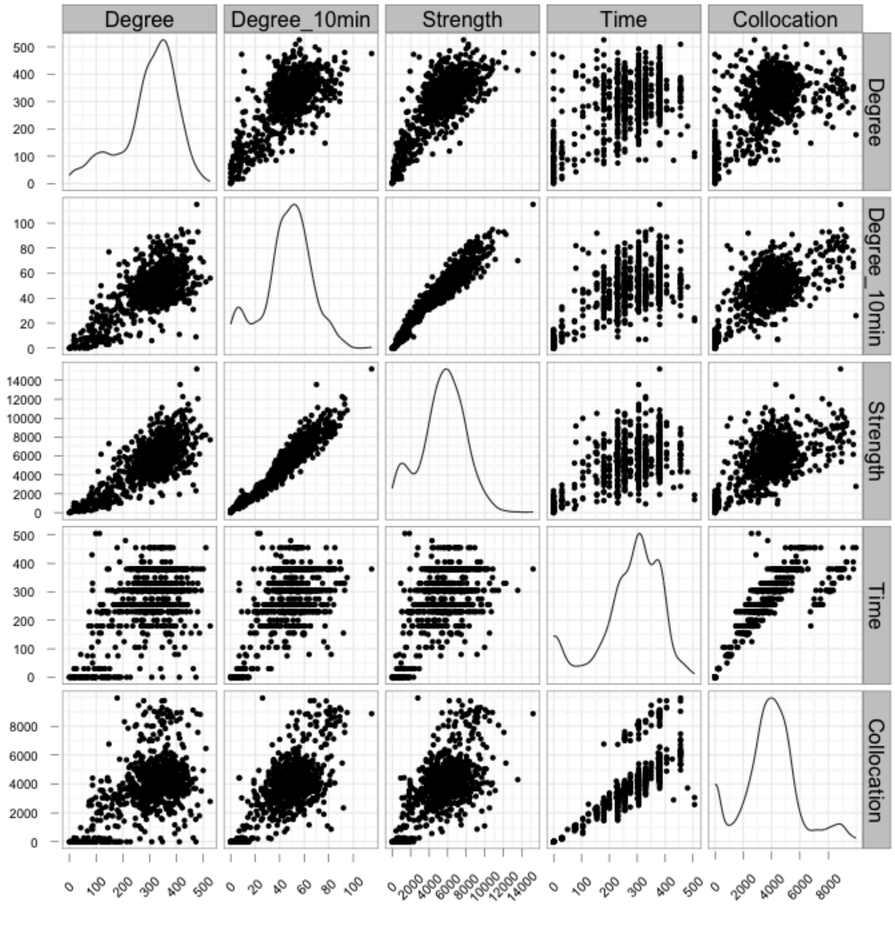

Fig. S9: The scatterplot matrix shows the relationships between all five indicators used in the paper. The matrix diagonal contains density plots of all indicators.

Collocation indicator versus the individuals' impact as index case

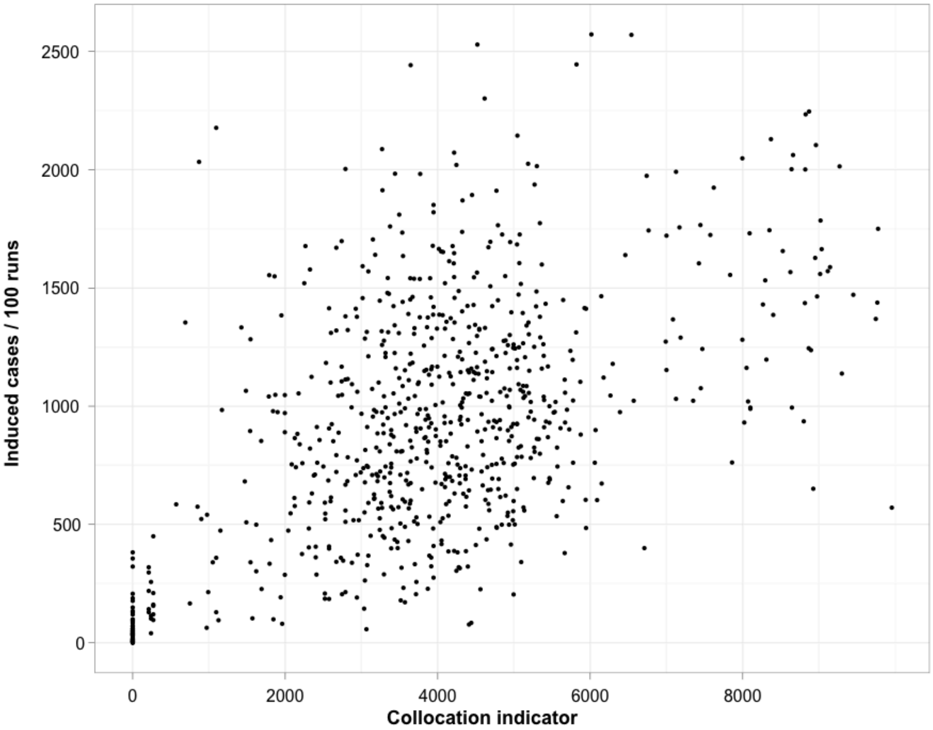

Fig. S10: Scatterplot of the individuals' collocation indicator values versus the number of infections they induced in those 100 simulation runs for which the respective individual was the index case.

References

1. Salathé M, Kazandjieva M, Lee JW, Levis P, Feldman MW, et al. (2010) A high-resolution human contact network for infectious disease transmission. Proc Natl Acad Sci U S A 107: 22020–22025.

2. Jaccard P (1901) Étude comparative de la distribution florale dans une portion des Alpes et des Jura. Bull Soc Vaudoise Sci Nat 37: 547–579.
